# The effect of incorrect scanning distance on boundary detection errors and macular thickness measurements by spectral domain optical coherence tomography: a cross sectional study

**DOI:** 10.1186/1471-2415-14-148

**Published:** 2014-11-27

**Authors:** Boglárka Enikő Varga, Erika Tátrai, Delia Cabrera DeBuc, Gábor Márk Somfai

**Affiliations:** Department of Ophthalmology, Faculty of Medicine, Semmelweis University, 39 Mária Str., Budapest, 1085 Hungary; Bascom Palmer Eye Institute, University of Miami, Miller School of Medicine, 1638 NW 10th Avenue, Miami, FL 33136 USA

**Keywords:** Optical coherence tomography, Image segmentation, Imaging pitfalls, Diabetic retinopathy, Macular degeneration

## Abstract

**Background:**

To investigate the influence of scan distance on retinal boundary detection errors (RBDEs) and retinal thickness measurements by spectral domain optical coherence tomography (SD-OCT).

**Methods:**

10 eyes of healthy subjects, 10 eyes with diabetic macular edema (DME) and 10 eyes with neovascular age-related macular degeneration (AMD) were examined with RTVue SD-OCT. The MM5 protocol was used in two consecutive sessions to scan the macula. For the first session, the device was set 3.5 cm from the eye in order to obtain detectable signal with low fundus image quality (suboptimal setting) while in the second session a distance of 2.5 cm was set with a good quality fundus image. The signal strength (SSI) value was recorded. The score for retinal boundary detection errors (RBDE) was calculated for ten scans of each examination. RBDE scores were recorded for the whole scan and also for the peripheral 1.0 mm region. RBDE scores, regional retinal thickness values and SSI values between the two sessions were compared. The correlation between SSI and the number of RBDEs was also examined.

**Results:**

The SSI was significantly lower with suboptimal settings compared to optimal settings (63.9±12.0 vs. 68.3±12.2, respectively, p = 0.001) and the number of RBDEs was significantly higher with suboptimal settings in the “all-eyes” group along with the group of healthy subjects and eyes with DME (9.1±6.5 vs. 6.8±6.3, p = 0.007; 4.4±2.6 vs. 2.5±1.6, p = 0.035 and 9.7±3.3 vs. 5.1±3.7, p = 0.008, respectively). For these groups, significant negative correlation was found between the SSI and the number of RBDEs. In the AMD group, the number of RBDEs was markedly higher compared to the other groups and there was no difference in RBDEs between optimal and suboptimal settings with the errors being independent of the SSI. There were significantly less peripheral RBDEs with optimal settings in the “all-eyes” group and the DME subgroup (2.7±2.6 vs. 4.2±2.8, p = 0.001 and 1.4±1.7 vs. 4.1±2.2, p = 0.007, respectively). Retinal thickness in the two settings was significantly different only in the outer-superior region in DME.

**Conclusions:**

Optimal distance settings improve SD-OCT SSI with a decrease in RBDEs while retinal thickness measurements are independent of scanning distance.

## Background

Optical coherence tomography (OCT) is one of the most important decision making technologies used in ophthalmology [1]. Cross-sectional OCT images of the retina correlate well with retinal histology [2–4] and can be used for quantitative analysis of retinal morphology, i.e. measurement of retinal thickness [5]. This quantitative analysis can help the follow-up of several retinal pathologies [6, 7] and facilitates important clinical decisions, as in the case of diabetes [8–10] or age-related macular degeneration (AMD) [11]. The latest spectral domain OCT (SD-OCT) technology has enabled a substantially increased sampling speed providing more detailed retinal imaging and fewer artifacts [12, 13].

The retinal thickness measurements (RTMs) of OCT devices are based on segmentation algorithms that delineate the vitreo-retinal interface and the outer retina, with the latest devices being able to quantify intraretinal structure by retinal layer segmentation. It is well known that this segmentation is prone to artifacts both in healthy eyes [14–17] and in eyes with macular pathologies [18–22] which might severely influence thickness measurements and might bias clinical decisions. Furthermore, the quantitative analysis of retinal morphology by segmentation algorithms is also sensitive to image quality [17, 23]. Recent evidence indicates that the variability of scan quality could predict retinal boundary detection errors (RBDEs) [24, 25]. We have previously shown the comparability of the segmentation of time domain OCT images with those obtained by RTVue SD-OCT (Optovue Inc., Fremont, CA, USA), observing some regional differences being difficult to explain [26]. As the RTVue device - similarly to other commercially available OCT devices – provides an automatic optimization process for the OCT settings (including focusing, polarization and Z-offset) that was employed in our study, a possible explanation for the differences was the suboptimal scan distance setting of the SD-OCT device, that is, the possibly longer than optimal scanning distance resulting in poor image quality and thus leading to measurement artifacts.

The aim of this study was to investigate whether scan distance settings of the RTVue SD-OCT have any influence on the errors in retinal boundary detection and RTMs in healthy eyes and eyes with retinal pathologies such as diabetic macular edema (DME) and AMD.

## Methods

Ten eyes of ten healthy subjects, ten eyes of ten patients with DME and ten eyes of ten patients with neovascular AMD were included in our study. Patients were recruited consecutively from our Retina Clinic. The study eye was selected randomly if both eyes were eligible for the study. The study was approved by the Semmelweis University Regional and Institutional Committee of Sciences and Research Ethics. All patients gave informed consent to the study and were treated according to the tenets of the Declaration of Helsinki.

**Figure 1:**
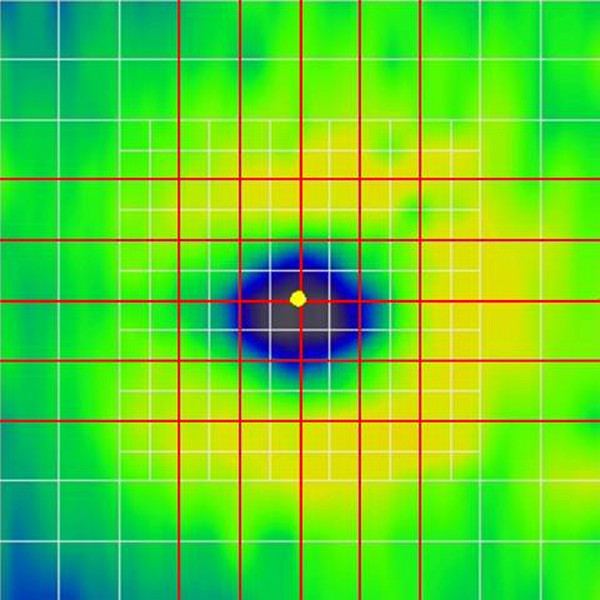
**Macular map showing the scanning lines of the RTVue MM5 protocol.** The protocol consists of 34 line scans (white and red lines). The red lines indicate the scans that were chosen for analysis, each being 5 mm in length.

Each subject underwent routine ophthalmic examination including best corrected visual acuity measurement, applanation tonometry and slit-lamp examination. All study subjects were assessed by the same, expert and trained operator with an RTVue OCT device using MM5 macular scan protocol under non-mydriatic circumstances. The MM5 protocol makes image of an 5 × 5 mm area with 11 horizontal and 11 vertical B-scans composed of 668 A-scans each and an inner 3 × 3 mm area with 6 horizontal and 6 vertical B-scans composed of 400 A-scans each (see Figure 1).

**Figure 2:**
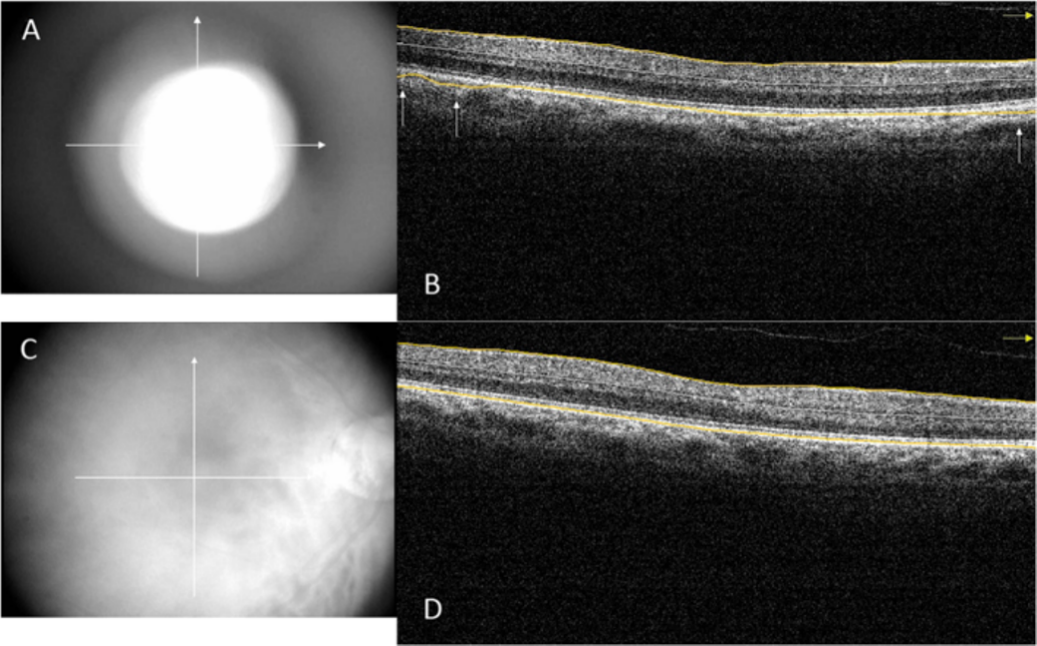
**Fundus and macular OCT images taken with suboptimal and optimal settings with RTVue OCT system.** Inner and outer retinal segmentation lines are highlighted with yellow color for better observation. Errors are signed with white arrows. **(A)** Fundus image taken with suboptimal settings in a healthy subject. Note the concentric narrowing of the fundus image due to the pupillary border decreasing the field of view. **(B)** Corresponding OCT scan of **A)** fundus image. (Signal strength index =66.8) Outer retinal misidentification is observable at the peripheral region of the scan. **(C)** Fundus image taken with optimal settings in the same healthy subject. Note that the fundus image fills the entire image. **(D)** Corresponding OCT scan of **C)** fundus image. (Signal strength index =72.4) The scan does not contain any errors.

**Figure 3:**
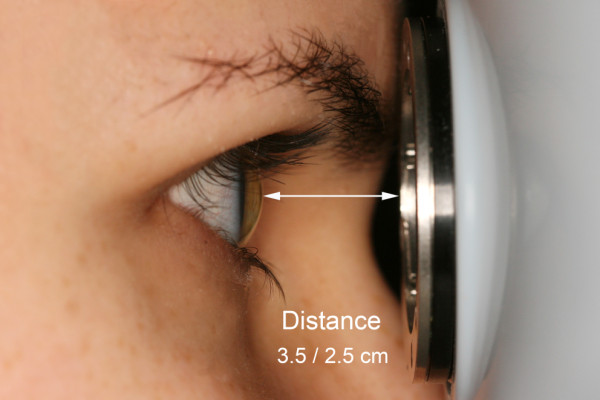
**The measurement of the scanning distance between the anterior surface of the cornea and the imaging lens.**

Optimal OCT images require not only optimal focus, polarization and Z-offset settings but also a best-possible fundus image is necessary in order to avoid errors due to peripheral artifacts because of the aperturing effect of the pupil. Therefore, we empirically measured the distance of the OCT device when good quality fundus images were obtained with the view of the fundus visible in the entire fundus image window (see Figure 2C) and found this distance to be approximately 2.5 cm. The distance was measured from the side between the surface of the centre of the cornea and the imaging lens of the device (Figure 3). For the first session, the device was set at 3.5 cm from the eye in order to obtain detectable signal with low fundus image quality with peripheral obscuring of the fundus (suboptimal scan distance setting, see Figure 2A) while in the second session a distance of 2.5 cm was set with a good quality fundus image (optimal scan distance setting). A minimum of 5 minutes elapsed between the two sessions. For both sessions the scan settings were optimized using the built-in optimization option of RTVue before taking all scans.

The RTVue OCT defines the signal strength index (SSI) as the average signal strength of the OCT scan, its value ranging from almost 0 to 90 (no detectable signal to best signal strength). Because of the observable correlation between SSI and the quality of the OCT scan, this parameter was used as indicator of the image quality in our study.

Signal strength index (SSI) and regional thickness values were recorded. The score for inner and outer RBDE was calculated for five vertical and five horizontal selected scans of the MM5 grid protocol from each eye for both settings according to a grading system based on the scoring previously described by Sadda et al. [14] and modified for the current study (Figure 1 and Table 1). A total of 600 scans were examined by an operator, who was blinded for the group and the setting of the images. The final score for each scanned eye was the sum of the scores of the 10 scans; therefore, the higher the score number, the more errors are present. In order to assess segmentation errors at the periphery, RBDE scores were also recorded and calculated similarly for the peripheral regions 1.0 mm from the horizontal scan edge on both sides on all scans (Figure 2). The number of errors in the remaining central region was defined as the difference between the total number of errors found in the whole scan and the number of peripheral errors.

**Table 1:** **Scoring system used for the assessment of retinal boundary detection errors**

1 point	Any boundary detection error (deviation of at least 4×10 pixels [height x width])
1 point	Any boundary detection error within the area of the fovea (central 1 mm of the 5 mm long scan)
1 point	The sum of horizontal errors exceeds 1 mm
1 point	The sum of horizontal errors exceeds 3 mm
1 point	The sum of vertical errors exceeds 1/3 of total retinal thickness
1 point	The sum of vertical errors exceeds 1/3 of total retinal thickness

When retinal boundary detection errors were present in both the inner and outer retinal boundaries, the errors were summed to assess the severity of the axial or transverse error.

The correlation between the SSI and the number of RBDEs was examined using linear correlation including all scans taken both with suboptimal and optimal scan distance settings. SSI values, RBDE scores and regional retinal thickness values were compared between the two sessions using Wilcoxon test. Intraclass correlation coefficients (ICC) with 95% confidence intervals were calculated for these variables, followed by the direct comparison of confidence intervals These analyses were performed for all participating eyes (“all-eyes” group) and for the three subgroups (normal, DME and AMD groups). The statistical analyses were performed with Statistica 8.0 (Statsoft Inc., Tulsa, OK, USA) and SPSS 19 (IBM Corp., Armonk, NY, USA) softwares. The level of significance was set at 5%.

## Results

The SSI was significantly lower with suboptimal scan distance settings compared to optimal scan distance settings (63.9 ± 12.0 vs. 68.3 ± 12.2, respectively, p = 0.001). The number of RBDEs was significantly lower with optimal scan distance settings in the “all-eyes” group for the entire scan and also for the peripheral but not the central scan parts (Table 2). The number of RBDEs negatively correlated with the SSI value (Table 3). The ICC of the RBDE scores of the two settings showed a mild difference, except the peripheral region where it was low (Table 4).

**Table 2:** **Retinal boundary detection error scores obtained with suboptimal and optimal scan distance settings**

	“All-eyes” group			Normal group		
RBDEs	Suboptimal setting	Optimal setting	p	Suboptimal setting	Optimal setting	p
Entire scan	***9.1 ±6.5***	***6.8 ±6.3***	***0.007***	***4.4 ±2.6***	***2.5 ±1.6***	***0.036***
	***8 [4, 11]***	***5 [2, 11]***		***4 [3, 6]***	***2 [1, 4]***	
Center	4.8 ±5.2	4.1 ±4.7	0.225	1.2 ±1.7	0.7 ±1.1	0.285
	4 [1,7]	3 [0, 7]		1 [0, 2]	0 [0, 1]	
Periphery	***4.2 ±2.8***	***2.7 ±2.6***	***0.001***	3.2 ±2.4	1.8 ±1.3	0.093
	***4 [3, 5]***	***2 [1, 4]***		4 [2, 4]	2 [1, 3]	
	**DME group**			**AMD group**		
**RBDEs**	**Suboptimal setting**	**Optimal setting**	**p**	**Suboptimal setting**	**Optimal setting**	**p**
Entire scan	***9.7 ±3.3***	***5.1 ±3.7***	***0.008***	13.1 ±8.7	12.9 ±6.8	0.919
	***10 [8, 11]***	***5 [3, 8]***		14 [5, 18]	14 [10, 17]	
Center	5.6 ±3.6	3.7 ±2.8	0.097	7.7 ±6.8	7.9 ±5.7	0.833
	6 [4, 7]	4 [2, 5]		6 [2, 14]	9 [3, 13]	
Periphery	***4.1 ±2.2***	***1.4 ±1.7***	***0.008***	5.4 ±3.5	5.0 ±2.8	0.441
	***4 [3, 5]***	***1 [0, 3]***		5 [3, 9]	6 [2, 7]	

The results for the pitfalls are shown as means ± SD and median [interquartile range]. Bold and italic font is used for the indication of significant differences between groups. Retinal boundary detection error (RBDE) scores and regional retinal thickness values between the suboptimal and optimal scan distance settings were compared using Wilcoxon test.

*Abbreviations*: *RBDEs* retinal boundary detection error scores, *DME* diabetic macular edema, *AMD* age-related macular degeneration, *SSI* signal strength index, *SD* standard deviation.

**Table 3:** **The correlation between the SSI and RBDE scores**

	“All-eyes” group		Normal group		DME group		AMD group	
	r	p	r	p	r	p	r	p
Entire scan	***-0.47***	***<0.001***	***-0.61***	***0.004***	***-0.57***	***0.009***	0.09	0.704
Center	***-0.44***	***<0.001***	***-0.69***	***0.001***	***-0.47***	***0.035***	0.10	0.674
Periphery	***-0.31***	***0.017***	-0.23	0.329	-0.35	0.136	0.02	0.921

The Spearman correlation coefficients (r) and corresponding p values are shown. Bold and italic font is used for the indication of significance.

*Abbreviations*: *SSI* signal strength index, *RBDE* retinal boundary detection errors, *DME* diabetic macular edema, *AMD* age-related macular degeneration.

**Table 4:** **Intraclass correlation coefficients (ICC) of the retinal boundary detection error scores and signal strength indexes (SSI) by study groups**

	All eyes	Normal	DME	AMD
Entire	0.718	0.337	0.281	0.724
	[0.445; 0.862]	[-0.152; 0.756]	[-0.132; 0.714]	[0.191; 0.924]
Center	0.766	0.476	0.432	0.752
	[0.568; 0.881]	[-0.137; 0.835]	[-0.120; 0.810]	[0.252; 0.933]
Periphery	0.591	0.206	0.253	0.889
	[0.187; 0.803]	[-0.297; 0.690]	[-0.131; 0.689]	[0.632; 0.971]
SSI	0.814	0.941	0.670	0.316
	[0.488; 0.923]	[0.580; 0.987]	[0.141; 0.905]	[-0,176; 0.745]

The results of the reliability examination of the RBDE scores between optimal and suboptimal scan distance settings are shown as ICC and the lower and upper border of the 95% confidence intervals.

*Abbreviations*: *ICC* Intraclass correlation coefficients, *RBDE* retinal boundary detection error, *SSI* signal strength index, *DME* diabetic macular edema, *AMD* age-related macular degeneration.

**Figure 4:**
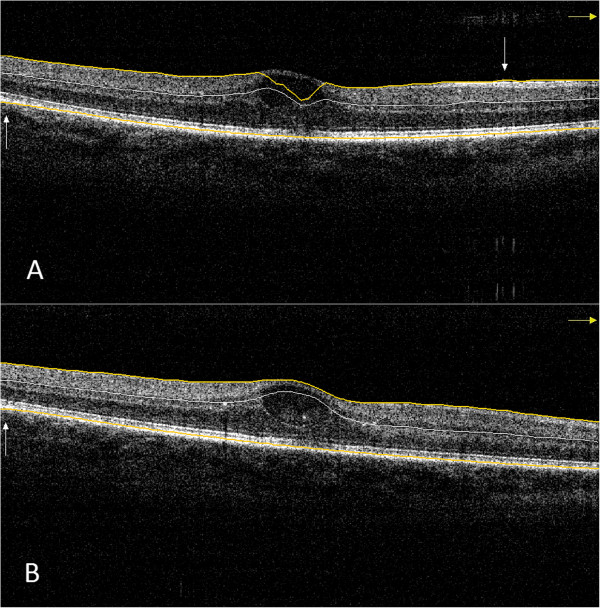
**Macular OCT scan of a subject with diabetic macular edema. (A)** Image taken with suboptimal settings. (Signal strength index =70.4). Inner retinal misidentification is observable in the central region and outer retinal misidentification at the peripheral region of the scan. **(B)** Image taken with optimal settings. (Signal strength index =74.3). Outer retinal misidentification is observable at the peripheral region of the scan. Inner and outer retinal segmentation lines are highlighted with yellow color for better observation. Errors are signed with white arrows.

**Figure 5:**
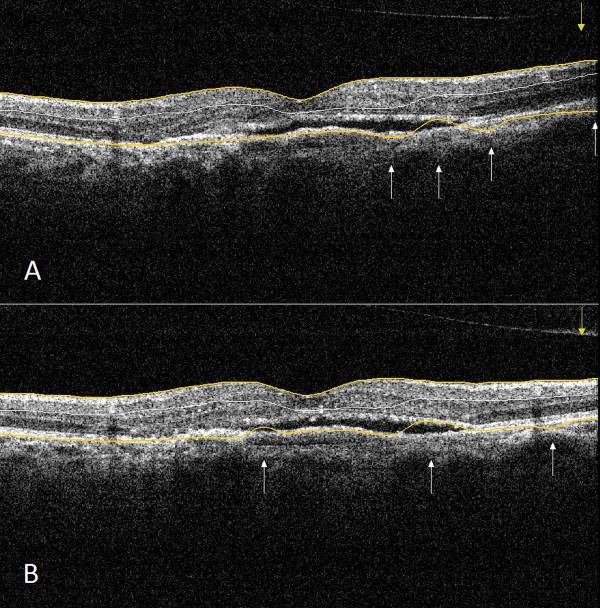
**Macular OCT scan of a subject with age-related macular degeneration. (A)** Image taken with suboptimal settings. (Signal strength index =57.0) Outer retinal misidentification is observable at the paramacular and the peripheral region of the scan. **(B)** Image taken with optimal settings. (Signal strength index =75.0) Outer retinal misidentification is observable in the central and at the paramacular region of the scan. Inner and outer retinal segmentation lines are highlighted with yellow color for better observation. Errors are signed with white arrows.

In the case of the normal group, the number of RBDEs was significantly lower with the optimal scan distance setting only for the entire scan, while in the case of the DME group the same trend was observed both for the entire scan and the peripheral scan parts (Figure 4 and Table 2). However, the ICC indicated a high difference between the two settings in these groups in all scan parts. Moreover, the SSI values showed a mild but significant negative correlation with the number of RBDEs along the entire scan and its central parts for both the normal and DME groups (Table 3). The ICC showed a mild difference in SSI values in DME but not in the normal group (Table 4). In the AMD group, the number of RBDEs was higher compared to all other groups and there was no difference in RBDEs between optimal and suboptimal settings, with the errors being independent of the SSI (Figure 5). This finding was supported by the ICC values, which indicated higher concordance between the two scan sessions than what was found in the other groups. The ICC showed the highest difference in SSI values between the two scanning sessions in eyes with AMD.

Regional retinal thickness measurements (RTMs) between the two scan distance settings were significantly different only in the inner and outer-superior region (R2 and R6, respectively) in the case of DME eyes and in R6 region in “all-eyes” group (see Table 5). There was a high correlation between the RTM values of the two sessions in each region (see Table 6).

**Table 5:** **The regional retinal thickness values obtained with suboptimal and optimal scan distance settings**

	“All-eyes” group			Normal group		
ETDRS regions	Suboptimal setting (μm)	Optimal setting (μm)	p	Suboptimal setting (μm)	Optimal setting (μm)	p
R1	295.7 ±137.7	301.0 ±139.5	0.100	242.5 ±17.8	242.1 ±17.1	0.208
R2	340.7 ±78.9	342.2 ±85.8	0.072	327.3 ±10.4	326.8 ±12.4	1.000
R3	338.9 ±95.9	348.6 ±103.3	0.106	323.3 ±17.9	320.7 ±13.8	0.063
R4	335.4 ±78.8	349.3 ±95.4	0.178	323.1 ±15.7	327.2 ±13.8	0.944
R5	338.1 ±98.6	348.7 ±102.4	0.734	309.8 ±12.0	316.1 ±13.6	0.919
R6	***303.9 ±54.9***	***303.6 ±55.5***	***0.023***	289.9 ±9.6	285.7 ±15.3	0.735
R7	319.7 ±64.3	304.3 ±59.4	0.627	306.1 ±10.7	285.0 ±8.8	0.445
R8	293.6 ±49.7	293.3 ±54.4	0.280	282.5 ±11.1	279.6 ±12.0	0.183
R9	305.1 ±61.8	313.0 ±65.8	0.290	284.5 ±9.8	282.8 ±10.0	0.066
	**DME group**			**AMD group**		
**ETDRS regions**	**Suboptimal setting (μm)**	**Optimal setting (μm)**	**p**	**Suboptimal setting (μm)**	**Optimal setting (μm)**	**p**
R1	400.9 ±196.5	419.6 ±195.0	0.541	243.7 ±36.8	247.3 ±37.3	0.173
R2	***398.5 ±108.7***	***405.8 ±122.7***	***0.042***	296.4 ±35.7	298.8 ±34.8	0.477
R3	404.1 ±137.3	431.8 ±144.7	0.477	289.4 ±38.5	299.0 ±38.4	0.646
R4	397.2 ±104.0	428.1 ±131.2	0.646	285.8 ±32.8	298.2 ±34.5	0.097
R5	415.2 ±137.6	438.9 ±139.7	0.083	289.3 ±30.6	296.8 ±25.8	0.359
R6	***347.6 ±73.7***	***343.3 ±77.2***	***0.008***	274.3 ±23.4	284.1 ±31.1	0.314
R7	369.8 ±87.2	363.0 ±72.2	0.260	283.3 ±26.0	268.9 ±20.5	0.445
R8	336.4 ±59.2	341.3 ±70.6	0.919	261.9 ±28.9	262.3 ±21.7	0.477
R9	362.0 ±75.6	373.8 ±85.6	0.154	268.7 ±25.9	285.6 ±23.4	0.086

The thickness values are shown as means ± SD. Regional retinal thickness values between the suboptimal and optimal scan distance settings were compared using Wilcoxon test. Bold and italic font is used for the indication of significant differences between groups.

*Abbreviations*: *DME* diabetic macular edema, *AMD* age-related macular degeneration, *ETDRS* Early Treatment Diabetic Retinopathy Study.

**Table 6:** **Intraclass correlation coefficients (ICC) of the retinal thickness values by study groups**

	All eyes	Normal	DME	AMD
R1	0.995	0.978	0.994	0.973
	[0.990; 0.998]	[0.909; 0.995]	[0.979; 0.999]	[0.896; 0.993]
R2	0.988	0.983	0.986	0.992
	[0.975; 0.994]	[0.935; 0.996]	[0.942; 0.996]	[0.968; 0.998]
R3	0.991	0.990	0.992	0.940
	[0.981; 0.996]	[0.953; 0.997]	[0.970; 0.998]	[0.790; 0.985]
R4	0.988	0.989	0.983	0.991
	[0.976; 0.994]	[0.957; 0.997]	[0.938; 0.996]	[0.959; 0.998]
R5	0.998	0.925	0.998	0.974
	[0.995; 0.997]	[0.731; 0.981]	[0.993; 1.000]	[0.905; 0.993]
R6	0.992	0.978	0.990	0.980
	[0.982; 0.996]	[0.916; 0.995]	[0.946; 0.998]	[0.927; 0.995]
R7	0.992	0.924	0.996	0.931
	[0.984; 0.996]	[0.741; 0.980]	[0.985; 0.999]	[0.762; 0.982]
R8	0.866	0.953	0.771	0.976
	[0.739; 0.933]	[0.817; 0.988]	[0.212; 0.919]	[0.914; 0.994]
R9	0.995	0.927	0.993	0.991
	[0.990; 0.998]	[0.664; 0.983]	[0.973; 0.998]	[0.956; 0.998]

The results of the reliability examination of the retinal thickness values between optimal and suboptimal scan distance settings are shown as ICC and the lower and upper border of the 95% confidence intervals.

*Abbreviations*: *ICC* Intraclass correlation coefficients, *DME* diabetic macular edema, *AMD* age-related macular degeneration.

## Discussion

Retinal thickness measurements obtained by OCT are an important source of information for both diagnostic and therapeutic decisions in retinal pathologies like DME and AMD. However, the algorithms incorporated in the OCT software are prone to boundary detection errors which may severely influence RTM results [14, 19]. In the present work we examined the effect of distance settings on retinal segmentation and thickness measurements in healthy eyes and eyes with DME and AMD. We chose non-mydriatic imaging as the latest OCT devices do not require pupil dilation and thus we could simulate the real-life settings of a screening scenario where an operator with basic training is capturing images.

There were several studies published earlier about the error types and their frequencies in automatic macular thickness measurements with different devices and in cases of various retinal pathologies [27]. The possible OCT image artifact categories were published by *Ray et al.*[18] using a time-domain OCT device, from which we observed four: inner retinal misidentification, outer retinal misidentification, degraded image and “off-center” artifacts. In the case of SD-OCT novel artifacts have also been described lately: incomplete segmentation lines and no segmentation lines being placed along the inner or the outer retina [27, 28], but we did not see such artifacts in our study.

Recently, *Giani et al.* compared automatic RTM errors of six SD-OCT devices in healthy subjects and in eyes with various pathological conditions [19]. According to their results, RTVue did not make errors in the case of healthy subjects, but in eyes with neovascular AMD or cystoid macular edema errors were detected in 58.3% and 38.4%, respectively, with the majority of errors located in the central area [19]. Similarly, *Ho et al.* found that RTVue had clinically significant errors in the central parts of the scans in 69% in eyes with AMD and in 25% in eyes with DME [29]. Interestingly, other SD-OCT devices used in their study showed a lower error rate; however, a number of studies have found the high reproducibility of RTVue measurements [29–33].

*Han et al.* examined the type and frequency of image artifacts in two different SD-OCT devices in four disease groups and healthy subjects [28]. A significant difference in artifact frequency was observed between healthy eyes and pathologic cases, for most error types. In eyes with AMD the misidentification of the outer retinal border was more frequent than that of the inner, due to the disruption of the RPE and outer retinal layers, with a clinically significant error observed in 5.1% and 8% of the scans.

*Schneider et al.* examined RBDEs in Stratus OCT images in the eyes of patients with diabetic retinopathy [34]. Most of the artifacts were produced by hard exudates (41.5%), cystoid macular edema (31.7%) and fibrovascular proliferative tissue formation (17.0%) leading to the misidentification of retinal boundaries of the retina.

The outer RBDEs were examined by *Costa et al.* (2004) in different pathologic cases, among others in DME [16]. They found that there are two high density layers in the outer retinal boundary, the inner forming the line used in the thickness measurement. In the case of DME this layer was not detectable, leading to the segmentation artifacts, similarly to our results.

In a recent study, *Song et al*. examined segmentation and RBDEs in eyes without diseases and with retinal or subretinal diseases using SD-OCT [35]. In eyes with neovascular AMD segmentation errors occurred in 95.2% of the cases, and involved both the inner and outer boundaries. Inner RBDEs were also frequent in DME eyes (68%), while in normal eyes the frequency of segmentation errors was 30%. Errors in the central 1 mm region were examined with two protocols (12 macular scans and a 3D macular cube scan) in the three examination groups. Frequencies of central segmentation errors for the two protocols were the highest in the subretinal group (77.4% and 83.9% respectively) with less errors in the retinal group (67.7% and 68.9%, respectively), and normal subjects (27.5% and 22.5%, respectively).

In order to minimize the number of errors, optimal OCT settings are necessary, most of which can be automatically set by the OCT software. However, scanning distance plays an equally important role in obtaining a good quality scan mostly because of the effect of the pupil aperture due to the decreased field of view.

In our study we found significant differences in the number of boundary detection errors between optimal and suboptimal distance settings in healthy eyes and eyes with DME, while there was a high number of RBDEs regardless of the setting in AMD which is in accordance with previous reports. In our observation the errors were predominant in the peripheral regions of the macula. In the central region there was only a non-significant trend towards a lower error rate with optimal settings. For the entire scan length the errors were significantly less with optimal settings in the healthy and DME groups. Not surprisingly, signal strength did not correlate with the number of RBDEs in the case of AMD, which means that there is always a high number of errors regardless of the settings in OCT images obtained from patients with AMD. Interestingly, a significant correlation was observed between the SSI and the RBDE scores in the central but not the peripheral scan regions in the groups of healthy and DME eyes. When looking at all eyes in the study, the number of RBDEs was significantly influenced by the SSI both for the entire scan and separately for the central and peripheral parts.

Our results indicate that the effects of scanning distance on boundary detection errors are more predominant in cases where macular structure is less disrupted (i.e. healthy normal eyes and DME), while high RBDE scores were obtained regardless of the scan distance setting and the SSI in eyes with AMD. Despite the number of RBDEs depending on the scanning distance, we found only minor differences in regional RTMs in all groups involved in the study, indicating that scan distance settings have only a moderate effect on regional RTMs. Even though the MM5 protocol of the RTVue has a higher scanning density compared to TD-OCT, its scanning pattern is not comprising the whole structure of the macula in detail. One possible explanation for our results (ie. the low thickness measurement error vs. a high number of RBDEs) may be mediated by the proprietary algorithms using interpolation to obtain the regional RTMs which could smooth out small measurement differences, rendering lower thickness errors in healthy eyes.

Although we did not assess the effect of RBDEs on the precision of follow-up measurements, it may be stipulated that images with more RBDEs may have lower reliability in the precise follow up of thickness changes. Both DME and AMD treatment strategies are in part relying on changes of the central subfield mean thickness which may highlight the importance of such measurement errors.

Despite the above results there are some shortcomings of the study. First, it is difficult to compare the methodology with other studies for the central area because of the different study setup. Second, this research was performed with one device, while other devices may have different error characteristics and therefore are results should be generalized with caution. Moreover, although the error grading and scoring was strictly defined, subjective factors could influence the final results. In addition, we involved a relatively low number of patients; however, the study size is comparable to other similar reports in the field. It should also be emphasized that OCT scan grading is time consuming and altogether 600 scans were analyzed for the study, with further attention to central or peripheral error location. Another potential limitation is that our study was based on non-mydriatic images as we were aiming at the simulation of a screening procedure. It has been shown previously that mydriasis is not affecting RNFL thickness measurements significantly, therefore we believe it did not add significant bias to our study [36]. Finally, we could have involved other pathologies for the comparisons, e.g. epiretinal membranes or retinal dystrophies, but DME and AMD are the two most important pathologies where OCT may play a crucial decision making role.

## Conclusions

To the best of our knowledge, this is the first study examining the effect of SD-OCT scan distance on image segmentation and retinal thickness measurements both in the central and peripheral locations of OCT scans. Retinal thickness measurements were proven to be robust in the central scan regions, not being influenced by the scanning distance and the accordingly different image quality. Despite this we believe that optimal distance settings are mandatory in order to obtain reliable results by SD-OCT.

## Abbreviations

AMD: Age-related macular degeneration
DME: Diabetic macular edema
OCT: Optical coherence tomography
RBDE: Retinal boundary detection error
RTM: Retinal thickness measurement
SSI: Signal strength index
SD-OCT: Spectral domain optical coherence tomography.
